# Theoretical study of the properties of X-ray diffraction moiré fringes. I

**DOI:** 10.1107/S2053273315004970

**Published:** 2015-05-14

**Authors:** Jun-ichi Yoshimura

**Affiliations:** a Photon Factory, Institute of Materials Structure Science, High Energy Accelerator Research Organization, 1-1 Oho, Tsukuba, Ibaraki 305-0801, Japan

**Keywords:** diffraction moiré fringes, *Pendellösung* oscillation, phase jump, gap phase

## Abstract

A detailed and comprehensive theoretical description of X-ray diffraction moiré fringes for a bicrystal specimen is given on the basis of a calculation by plane-wave dynamical diffraction theory. Firstly, prior to discussing the main subject of the paper, a previous article [Yoshimura (1997 ▸). *Acta Cryst.* A**53**, 810–812] on the two-dimensionality of diffraction moiré patterns is restated on a thorough calculation of the moiré interference phase. Then, the properties of moiré fringes derived from the above theory are explained for the case of a plane-wave diffraction image, where the significant effect of *Pendellösung* intensity oscillation on the moiré pattern when the crystal is strained is described in detail with theoretically simulated moiré images. Although such plane-wave moiré images are not widely observed in a nearly pure form, knowledge of their properties is essential for the understanding of diffraction moiré fringes in general.

## Abstract 2

A detailed and comprehensive theoretical description of X-ray diffraction moiré fringes for a bicrystal specimen is given on the basis of a calculation by plane-wave dynamical diffraction theory, where the effect of the *Pendellösung* intensity oscillation on the moiré pattern is explained in detail.

## 1. Introduction   

Crystal diffraction moiré fringes were discovered by Mitsuishi *et al.* (1951 ▸) in an electron micrograph of a graphite crystal and have been actively studied in the field of electron diffraction (Hashimoto & Uyeda, 1957 ▸; Pashley *et al.*, 1957 ▸; Bassett *et al.*, 1958 ▸). In the X-ray field, Bonse & Hart (1965 ▸, 1966 ▸) observed moiré fringes with X-ray interferometers from a silicon crystal, Chikawa (1965 ▸, 1967 ▸) observed them with an epitaxically grown CdS crystal and Lang & Miuscov (1965 ▸) observed them from a quartz crystal with a crack. Many interesting observations of moiré fringes were then successively reported. Brádler & Lang (1968 ▸) and Lang (1968 ▸) reported excellent moiré fringes observed with superposed crystals (*i.e*. bicrystal) of silicon and of quartz, respectively. Hart (1972 ▸) demonstrated a full analysis of moiré dislocations in a moiré pattern produced with an X-ray interferometer. Simon & Authier (1968 ▸), Bonse *et al.* (1969 ▸) and Gerward (1973 ▸) reported moiré fringes observed in ion-implanted silicon crystals. Although Bonse *et al.* (1969 ▸) referred to their observed fringes as ‘translation fault’ fringes (Bonse & Hart, 1969 ▸), Ohler *et al.* (1997 ▸) later explained that they are essentially moiré fringes. Moiré fringes observed with a monolithic type bicrystal, prepared by making a saw cut in a single crystal, were reported by Hashizume *et al.* (1972 ▸) and Tanemura & Lang (1973 ▸). Moiré fringes observed in a quartz crystal having etch tunnels were reported by Iwasaki (1977 ▸). Following these early studies, Jiang *et al.* (1990 ▸), Prieur *et al.* (1996 ▸), Ohler *et al.* (1996 ▸, 1999 ▸) observed moiré fringes with SIMOX (separation by implanted oxygen) silicon wafers, and analysed and discussed them. In particular, Ohler *et al.* (1999 ▸) reported excellent moiré fringes taken in the geometry of the Bragg case. With a different aim from the above studies, Yoshimura (1989 ▸, 1991 ▸, 1996*a* ▸) experimentally observed a strange oscillation (non-projectiveness) of moiré fringes on the beam path after emerging from a specimen crystal.

Whereas experimental studies have been actively conducted, theoretical study of X-ray moiré fringes has not received enough attention. This is in contrast with the study of *Pendellösung* and related fringes, where good theoretical studies have been made from an early stage (*e.g.*, Kato, 1961*a* ▸,*b* ▸). The first theoretical description of diffraction moiré fringes was given for the case of electron diffraction by Hashimoto *et al.* (1961 ▸). However, this theory appeared much too complicated for a neophyte to apply it to the X-ray case. The first attempt to theoretically describe X-ray moiré fringes was made by Simon & Authier (1968 ▸), where diffracted waves carrying moiré interference from a bicrystal were expressed on the basis of the Takagi–Taupin theory (Takagi, 1962 ▸) to calculate the intensity of diffracted images. Tanemura & Lang (1973 ▸) theoretically described the bicrystal moiré interference on the basis of Kato’s spherical wave theory (Kato, 1961*a* ▸,*b* ▸). Furthermore, a theory of bicrystal moiré was also given by Bezirganyan & Aslanyan (1984*a* ▸,*b* ▸). Nevertheless, these theories were unsatisfactory in that the process of double diffraction producing the moiré interference was not described in detail, and the results did not appear to be readily applicable to other moiré observations.

In 1974, Kato published a diffraction theory for a crystal having a misfit boundary, where a change in the reciprocal-lattice vector 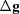 is induced between two parts of a single crystal as in a growth-sector boundary of crystals (Kato, 1974 ▸). It would not be too much to say that all theoretical preliminaries for dealing with double diffraction under 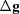 are given there. However, Kato did not proceed to describe moiré fringes. Polcarová (1978*a* ▸,*b* ▸, 1980 ▸), largely based on this theory by Kato, calculated diffraction intensities from a crystal having a misfit boundary, to a final form, but did not deal with moiré fringes. Yoshimura performed a full calculation of moiré fringes on the basis of Kato’s theory above, and has published part of the results as an appendix (Yoshimura, 1989 ▸, 1996*a* ▸) and a short note (Yoshimura, 1997*a* ▸). Although omitting to publish the entire results is regretted now, the full description would have had to be very long, and it was not the main investigative theme of the author at that time. Apart from Kato’s theory, Ohler & Härtwig (1999 ▸) published another theoretical description of bicrystal moiré fringes using a matrix formalism of dynamical diffraction (Berreman, 1976 ▸). Furthermore, Haroutyunyan & Sedrakyan (1997 ▸) also published a paper describing bicrystal moiré interference.

**Figure 1:**
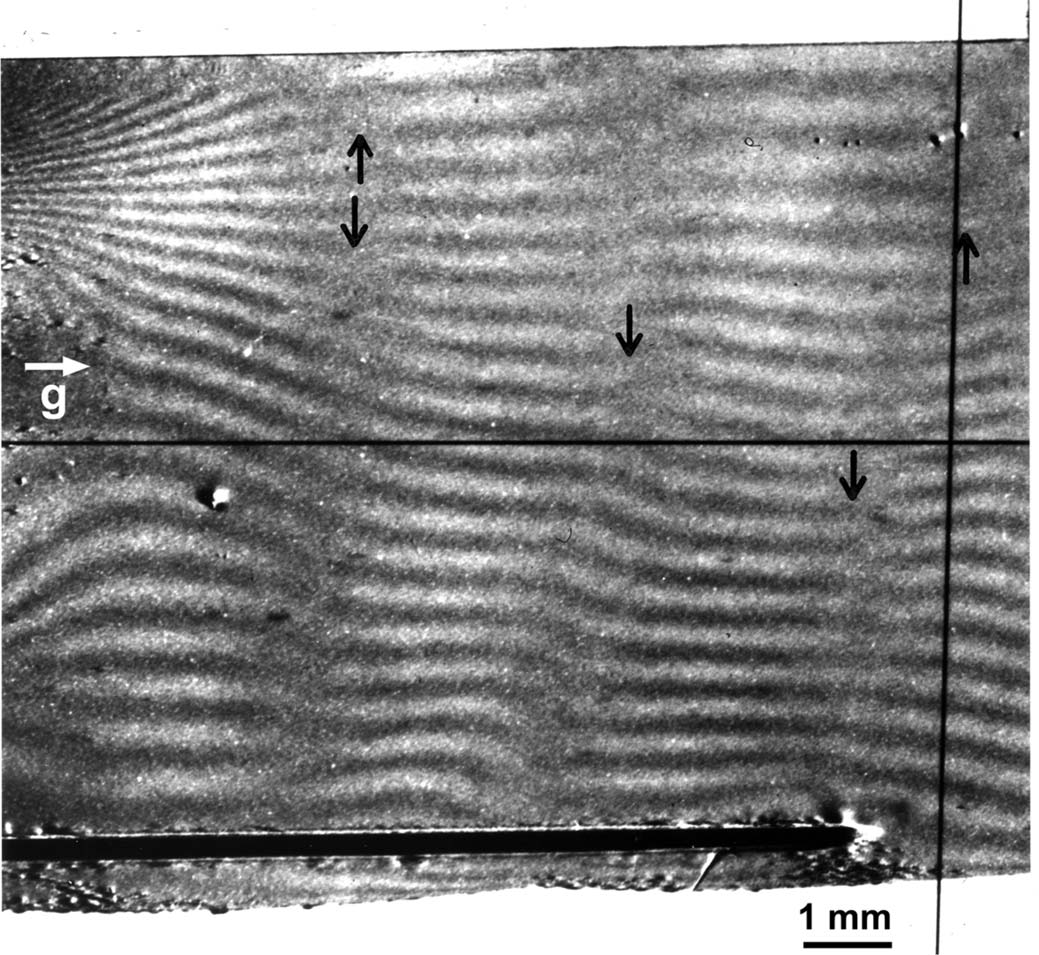
Experimental X-ray moiré image, the explanation of which was the starting point of this study. Diffracted-wave image (*G* image). Taken in a previous synchrotron experiment at PF, KEK, Japan (Yoshimura, 1996*a* ▸,*b* ▸), from a monolithic bicrystal specimen with Si 220 reflection and a wavelength of λ = 0.072 nm. The angular width of the incident beam was 0.34′′, and the total thickness of the bicrystal was 3.35 mm (including the gap thickness of 0.225 mm). The long vertical and horizontal lines are the shadows of a platinum line stretched between the specimen and the recording films for the purpose of the experiment. See text for more details.

The motive for the study leading to this paper is to give an explanation of the moiré image as shown in Fig. 1 ▸ (Yoshimura 1996*b* ▸, 1997*b* ▸), which was taken in a previous experiment on the moiré-fringe oscillation (Yoshimura, 1996*a* ▸). This moiré image, though nearly of rotation moiré, has a feature of low-contrast vertical bands extending from the top to the bottom of the image. Furthermore, the moiré fringes locally bend to a significant degree in these vertical bands, and fringe lines have dislocation-like discontinuities (noted by arrows) despite the absence of dislocations in the real lattice [called ‘pseudo-moiré dislocations’ in Yoshimura (1996*b* ▸)]. Such features were not observed in previously reported moiré images. They should also be explained for general interest. From the theoretical study of such experimental moiré images, it was found that *Pendellösung* intensity oscillation and the additional phase by an interspacing gap in the bicrystal have a significant effect on the moiré pattern. (In addition, the unusualness of this moiré pattern is considered to be related to the quasi-plane-wave condition when taking this image.) Although this work has been presented orally (*e.g*., Yoshimura, 2008 ▸), it has not been published as a paper.

The first purpose of writing this paper is to publish the above work. The second and main purpose is to present the theory of diffraction moiré fringes in a full form on the basis of Kato’s misfit-boundary diffraction theory. This will complete the author’s theoretical description of moiré fringes which has been given fragmentarily so far. Although the basic interest is in the above-mentioned experimental moiré images, the theory is described from a more general viewpoint. From the author’s experience, the description of moiré fringes is much more complicated than that of *Pendellösung* fringes and related images. To give a comprehensive description, treatment by plane-wave theory based on a schematic of the dispersion surface of diffraction would be a good approach. This paper will present such a treatment, with special attention on added phases by the *Pendellösung* intensity oscillation and by the interspacing gap as mentioned above. An exact and comprehensive description cannot help being long. Although diffraction moiré fringes are no longer a hot topic of study, the subject is still a branch of diffraction topography and crystallography. This paper may contribute to future progress in related research fields. In what follows, the moiré theory is first described from a general viewpoint, and then a theoretical explanation of the author’s previous moiré images by experiment is given. The work is divided into two separate publications, parts I and II.

## 2. Theoretical description of bicrystal moiré fringes   

**Figure 2:**
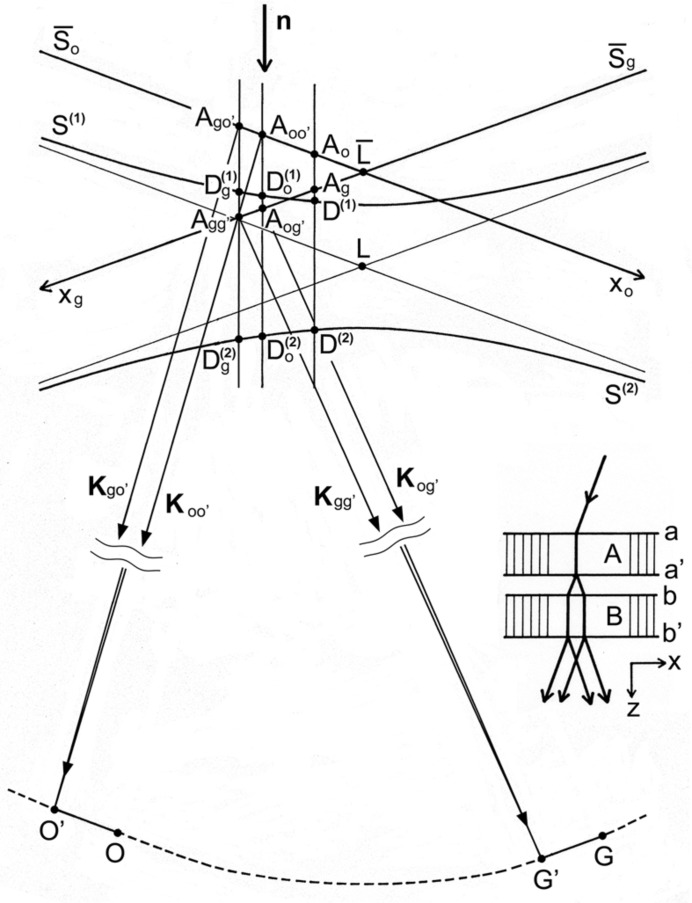
Dispersion-surface construction for the double diffraction in a bicrystal, with 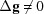. 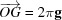, 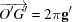. See text for details.

As a model for developing the theory, we consider a bicrystal as shown in Fig. 2 ▸, which is composed of parallel-sided crystals *A* and *B* having a difference 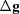 in their reciprocal-lattice vectors, and a narrow interspace gap between them. For simplicity, the surfaces of crystals *A* and *B* are all assumed to be parallel to one another, but the angle between the crystal surfaces and the diffracting lattice plane is taken to be arbitrary, so that the theory can deal with the asymmetric Laue case. If the dielectric susceptibility in crystal *A* having reciprocal-lattice vector 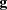 is given by 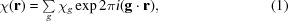
then that in crystal *B* having the reciprocal-lattice vector 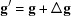 is written as 
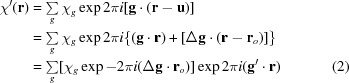
[because 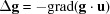]. Here, 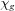 is the *g*-th Fourier component of 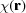 and 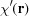; 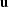 is the displacement in the real lattice of crystal *B* relative to that in crystal *A*, corresponding to the occurrence of 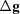; 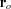 is the position vector denoting the point of 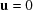 on surface *b* of crystal *B*. This origin 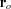 is not a very special point, but is explicitly written here for a later discussion.

The dispersion surface associated with the double diffraction of the moiré interference is shown in Fig. 2 ▸. By an incident plane wave 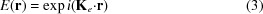
upon the bicrystal, transmitted (*O*) and diffracted (*G*) waves are first excited in crystal *A* (tie points *D* ^(1)^, *D* ^(2)^). As shown in Kato [1974 ▸, equations (4-105)–(4-108)], the excited *O* and *G* waves after emerging from crystal *A* are written and calculated as follows: 
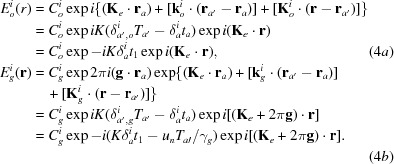
Here, 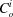 and 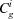 are the amplitudes of the excited waves, and are given as
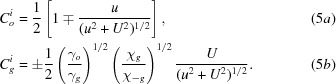


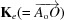
 is the wavevector of the initial incident wave; 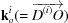 and 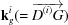 are the wavevectors of the excited waves propagating in crystal *A*, and 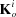 (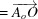) and 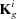 (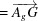) are those of the waves after emerging from crystal *A*. They are given by
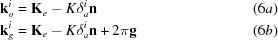


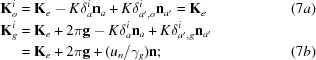

*K* in the above equations is the wavenumber in vacuum; the index 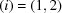 denotes the branch of the dispersion surface; 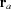 and 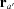 are position vectors denoting surfaces *a* and *a*′, and 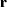 refers to an observation point thought to be situated behind the crystal; 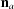 and 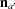 are the normals to surfaces *a* and *a*′, and are set equal to the common surface normal 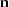, in accordance with the assumption in the present theory; 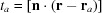 and 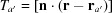; 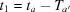 is the thickness of crystal *A*; 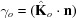 and 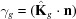; 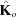 and 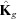 denote unit vectors along the directions of the transmitted and diffracted waves, respectively. 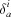, 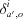 and 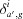 are the *Anpassung* associated with the excitation and emergence of waves in crystal *A*, and are graphically represented as 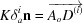, 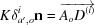, 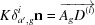 in Fig. 2 ▸; they are given in equation form by
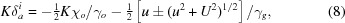


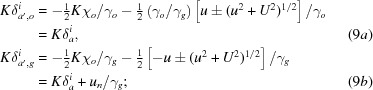

*u* in equations (5*a*), (5*b*), (8) and (9*a*), (9*b*) is the deviation parameter employed in this theory, for the excitation of *O* and *G* waves in crystal *A*; it is given by
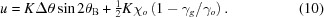


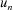
 in equations (4*b*), (7*b*) and (9*b*) is given by
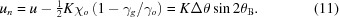
Here, 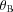 is the Bragg angle; 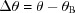 (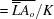 in Fig. 2 ▸) is the deviation angle from the exact Bragg position, θ being the incidence glancing angle to the diffracting lattice plane, on surface *a*; *U* is given by 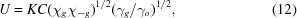
where *C* is the polarization factor. The relationships between the above deviation parameter *u* and those used in other literature, *W*, η *etc*. (Kikuta & Kohra, 1970 ▸; Authier, 2004 ▸) are 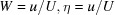.

Going back to equations (4*a*), (4*b*), each of the *O* and *G* waves emerging from crystal *A* excites another transmitted wave and diffracted wave on the incidence upon crystal *B*. Then the dispersion surface is displaced due to the change in the reciprocal-lattice vector from 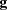 to 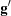, for each of the excitations by the *O* and *G* waves from crystal *A*. As treated in Kato (1974 ▸), the displaced dispersion surfaces are superposed onto that for crystal *A* so that the Lorentz point *L* is common; then, the reciprocal-lattice points *O*′ and *G*′ for crystal *B* are relocated consequently to the new positions so that 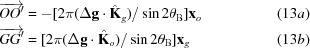
as shown in Fig. 2 ▸. The tie points for the secondly excited waves in crystal *B* are displaced as 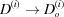 and 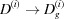 on the common dispersion surface. (It can easily be confirmed that the result of calculation using this scheme is the same as that when the displaced dispersion surfaces are given separately.) We denote the waves excited by the *O* wave as the (*O*, *O*′) and (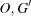) waves (tie points 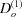, 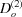), and those excited by the *G* wave as the (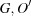) and (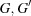) waves (tie points 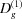, 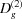). Following the way of the formulation of equations (4*a*), (4*b*), these doubly diffracted waves after emerging from crystal *B* can be written and calculated as follows: 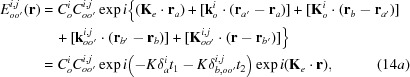


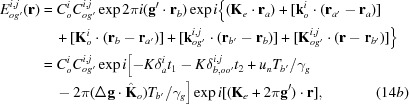


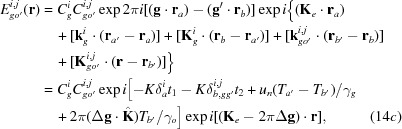


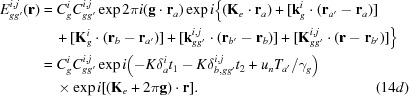
Here, 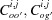 and 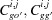 are the amplitudes for the secondly excited waves in crystal *B*, and are given by 
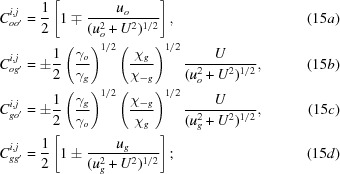
the indices (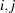) = (1, 2) denote the branch of the dispersion surface for crystals *A* and *B*, respectively; 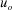 and 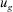 are the deviation parameters for the excitation of (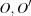), (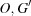) waves and (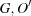), (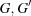) waves, respectively, and are given by 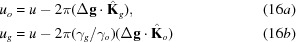
[see equations (5-68)–(5-71) in Kato (1974 ▸)]; 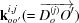, 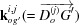, 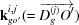 and 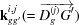 are the wavevectors of waves denoted by the respective superscripts and subscripts in crystal *B*, and 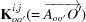, 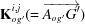, 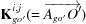 and 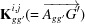 are the wavevectors after emergence from crystal *B*, which are given as follows:
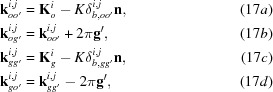


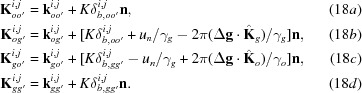
The *Anpassungs* 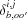 and 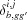 in the above equations are given in the form of *K* × *Anpassung*, as 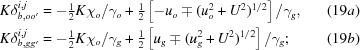


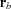
 and 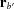 in equations (14*a*)[Disp-formula fd14a] [Disp-formula fd14b] [Disp-formula fd14c]–(14*d*)[Disp-formula fd14d] are the position vectors denoting surfaces *b* and 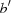; 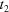 is the thickness of crystal *B*; 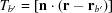.

Additionally, equation (16*b*) for 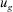 needs a detailed explanation. The displacement of the excited point on the 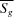 sphere (Fig. 2 ▸) from 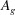 to 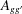 (corresponding to the displacement of the tie points from 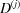 to 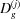) is given by 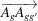 = 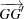 = 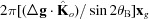. This is replaced by the corresponding displacement on the 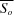 sphere 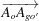 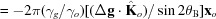, so that 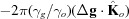 is added to the right-hand side of equation (16*b*) as its second term. Thus, 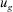 is given in the same measure as *u* and 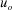, although it is associated with waves excited by the *G* wave. While *u* and 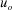 correspond to the deviation angle when the *O* wave strikes the (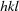) lattice plane, 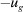 corresponds to the actual deviation angle with which the *G* wave strikes the (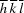) plane.

From the waves in equations (14*a*)[Disp-formula fd14a] [Disp-formula fd14b] [Disp-formula fd14c]–(14*d*)[Disp-formula fd14d], the intensity of the diffracted image from the bicrystal is calculated as follows (for brevity, only the intensity of the diffracted-wave image is shown): 
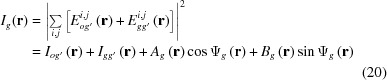
with 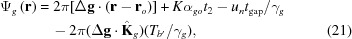


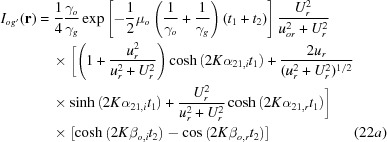


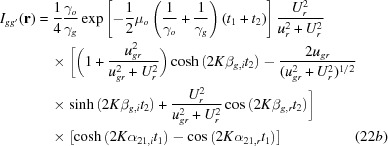


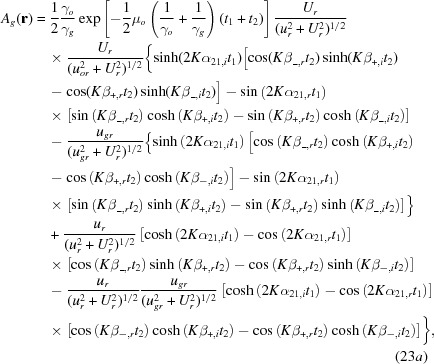


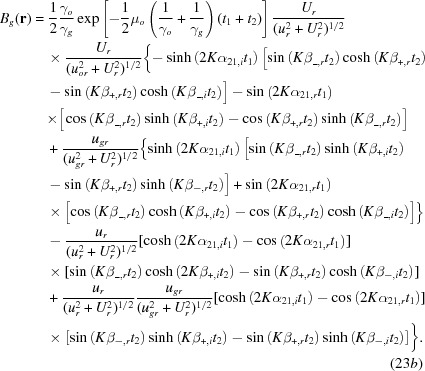
In this calculation, variables and constants *u*, *U* *etc*. in the amplitude parts are approximated by their real parts, and those in the phase parts are exactly calculated as complex quantities. The coherence coefficient is not considered. Indices *r* and *i* denote real and imaginary parts, respectively. 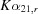, 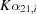 and 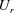, 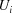 are given by 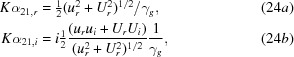


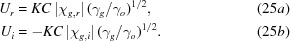


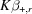
, 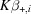 and 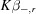, 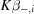 are the real and imaginary parts of the following quantities: 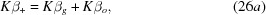


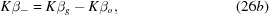


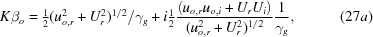


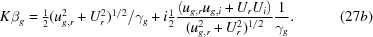


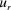
, 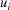, 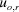, 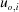 and 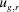, 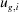 are given by 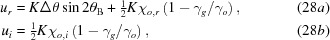


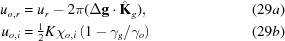


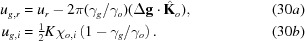


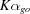
 is given by 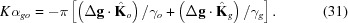
The symbol 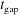 that appears in the phase term in equation (21) denotes the thickness of the interspacing gap between surfaces *a*′ and *b*, namely 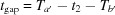; 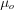 is the linear absorption coefficient for mean absorption.

## 3. Two-dimensionality of crystal diffraction moiré fringes   

**Figure 3:**
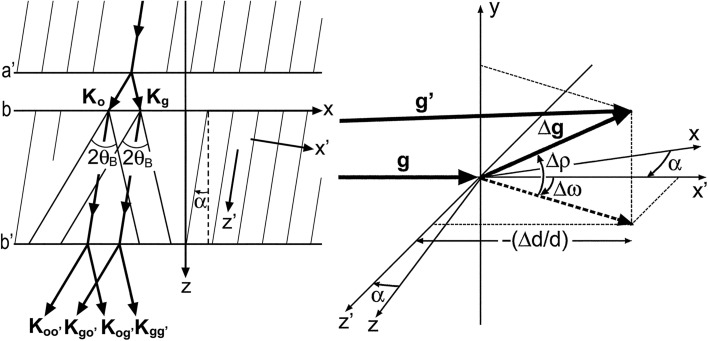
Coordinate systems in the discussion of the moiré phase, and graphical representation of components of 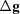. The two triangles in the left-hand side figure represent Borrmann fans for the incidence of the waves, 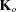 and 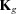. In this diagram 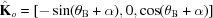 and 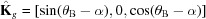.

The contents of the phase of the interference 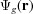 in the intensity field [equation (20)] can be further arranged. As has been shown in Yoshimura (1996*a* ▸), part of the first term and the fourth term in equation (21) for 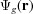 cancel each other. Noting that 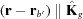, it turns out that 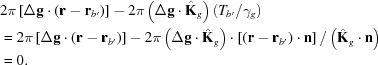
Furthermore, another part of the first term and the second term in equation (21) partially cancel each other (Yoshimura, 1997*a* ▸), as shown in the following. It can be seen immediately that the second term can be rewritten as 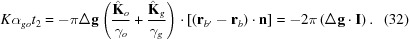
Here, 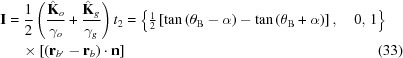
is the vector connecting the apex (on surface *b*) and the midpoint on the base (on surface *b*′) of the Borrmann fan which is supposed for dynamical diffraction in crystal *B* (see Fig. 3 ▸); 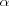 is the angle between the diffracting plane and the surface normal 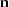. Thus, the second term can be decomposed as 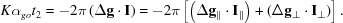
Here, the symbols 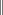 and 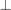 denote, respectively, the components parallel and perpendicular to surfaces *a*′ and *b*. On the other hand, the remaining part of the first term can be decomposed to
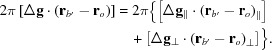
It was commented with equation (2) that the origin 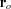 can be taken on surface *b*. Accordingly, the second terms on the right-hand side of these two equations cancel each other, namely 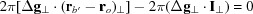, and the phase 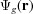 is reduced to 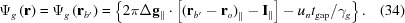
When the difference in the reciprocal-lattice vectors 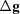 [defined with equation (2)] is written as 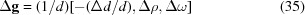
in the coordinate system 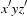, where the 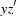 plane is on the diffracting lattice plane (see Fig. 3 ▸), 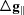 is given in the coordinate system with the *xy* plane taken on crystal surface *a*′ or *b*, by 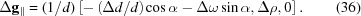
Here, *d* is the lattice spacing of the diffracting plane; 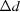 is the difference in *d*; 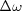 is the inclination of the diffracting plane about the *y* axis [parallel to 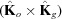]; 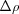 is the rotation about the axis perpendicular to 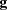 and 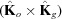.

Thus, the phase difference related to 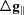, namely the intrinsic moiré phase, is written as 
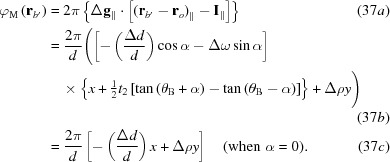
From equation (37*c*), the well known expressions for the spacing Λ and direction 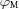 of moiré fringes are obtained: 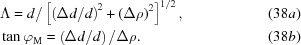
The calculation has so far been conducted in quite a general way. It may be stated here that the two-dimensional character of crystal diffraction moiré patterns, which arise from a three-dimensional vector 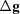, has been proved. Parallel moiré 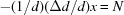 (*N* is an integer) and rotation moiré 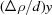 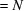 can occur, but the third type of moiré 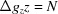 does not occur. However, the third component of 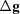, 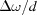, takes part in forming a parallel moiré pattern when 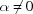.

**Figure 4:**
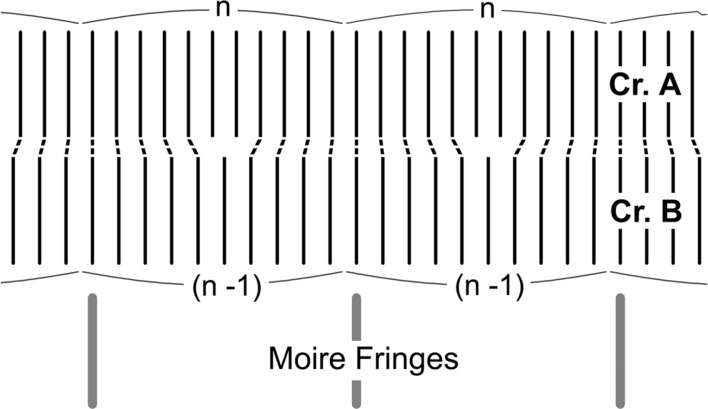
Schematic illustration to explain the generation of moiré fringes based on equation (37*c*) with Δρ = 0.

As seen from the above discussion, 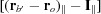 in equation (37*a*) is a vector on surface *b*. Therefore, the moiré phase 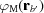 in equations (37*a*)–(37*c*) is practically determined on surface *b*. The observed moiré fringes are related to such a moiré phase. An experimental fact evidencing this point is that regarding moiré dislocations. As illustrated in Fig. 4 ▸, the moiré phase 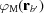 increases by 2π to add one moiré fringe, every site where the positions of two sets of lattice planes having a difference of 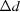 exactly coincide with each other. In this property, moiré fringes may be referred to as a counter of excess or deficient lattice planes. From this viewpoint, it can be well understood that, when a dislocation outcrops on one of the facing surfaces *a*′ or *b*, the moiré pattern sensitively responds to it to form a moiré dislocation. As Lang (1968 ▸) demonstrated, only such dislocations as outcropping on the inner facing surfaces give rise to moiré dislocations, and other dislocations do not affect the moiré pattern. Diffraction moiré fringes, though being interference fringes of light waves, produce a moiré pattern of superposed lattice planes by the same mechanism as geometrical moiré patterns. It is difficult to consider that a discontinuity in the lattice-plane arrangement such as that in Fig. 4 ▸ can occur on boundaries or surfaces other than those where the lattice cut actually occurs, as in a bicrystal and a cracked crystal. We now arrive at an inference that the absence of the lattice cut as above would be the reason why moiré fringes have not been found in diffraction images of growth-sector boundaries, despite the occurrence of 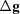. Discussion and analysis on moiré fringes agreeing with the above discussion of equation (37*a*) have also been given by Ohler *et al.* (1999 ▸). Although the discussion in this section has in substance been written in Yoshimura (1997*a* ▸), it was rewritten here to revive the previous remark on the two-dimensionality of the moiré pattern and to complete the moiré theory here.

## 4. Plane-wave image and effect of *Pendellösung* phase   

In this section, we present a theoretical simulation of several moiré-fringed diffraction images to demonstrate the property of moiré fringes. According to the results of the simulation work, it is when the angular width of the incident beam is approximately less than 0.02′′ that experimental moiré images given by the integrated intensity agree well with theoretical plane-wave moiré images. While all the experimental moiré images are obtained as an integrated intensity image in some measure, no moiré experiment has probably been made so far using such a highly collimated beam. In this sense, the property of a plane-wave moiré image appears in the pure form only under extreme experimental conditions. However, knowledge on the plane-wave moiré image would be useful in the study of diffraction moiré images in general, observed under wide experimental conditions.

The third and fourth terms in equation (20) for the moiré-image intensity, which involve the phase 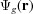, need to be unified to a single term for a discussion of the properties of interference images. Therefore, equation (20) is rewritten as 
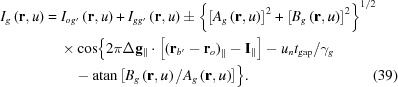
Here, 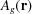 and 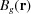 in equations (23*a*)[Disp-formula fd23a], (23*b*)[Disp-formula fd23b] are written as 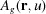 and 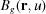 with regard to their dependence on the deviation parameter *u*; also 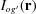 and 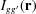 are similarly rewritten. The position vector 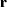 for the observation point may be taken to be the same as 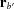 for the position on the exit surface *b*′, based on the projective property of the moiré image confirmed in equation (34) (in theory). The first term in the cosine function is written in the reduced form as given in equation (37*a*). The third, newly added term is related to the intensity oscillation due to the *Pendellösung* action in the crystal [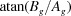 means 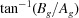]. Hence, the phase term is hereafter called the PL phase. The PL phase is a constant and does not significantly affect the fringe pattern, so far as the image intensity does not vary with the position in the image. However, when the crystal has some strain and crystal absorption for the beam intensity is small, this PL phase varies sensitively with 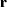 and *u*, to modify the intrinsic moiré pattern significantly. This oscillation is the same as the intensity oscillation called equi-inclination oscillation in a single crystal. Thus, two interferences of different origins, moiré and *Pendellösung* interferences, combine to make the one interference pattern of crystal diffraction moiré. Such an effect of the PL phase has already been described by Hashimoto *et al.* (1961 ▸). However, since then, not much attention has been paid to it in the X-ray field, until a remark by Yoshimura (1997*a* ▸). At this stage of the discussion on the plane-wave image, the gap phase 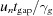 in the second term in the cosine function in equation (39) does not significantly affect the moiré-fringe pattern under discussion. However, it can affect the fringe pattern even in the plane-wave image, when the front crystal *A* is strained so that 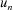 varies with the position in the crystal. The phase of the moiré interference is thus composed of the three terms as in equation (39). The phase term ‘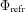 for refractive-index difference’ in Ohler & Härtwig (1999 ▸, p. 414) does not appear in the calculation of this paper. Furthermore, the above gap phase 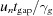 does not agree with their gap phase ‘
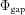
’.

**Figure 5:**
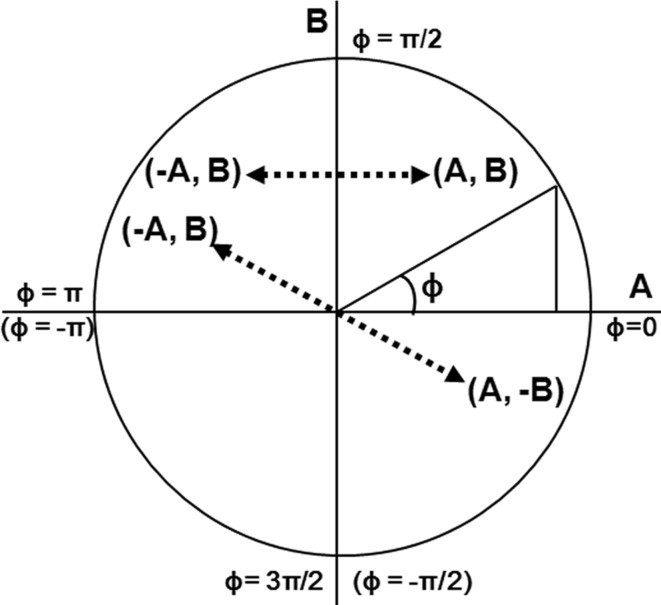
Quarter circle for explaining the phase jump of 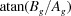, drawn in the plane of *A*–*B* coordinates.

One problem in expression (39) is the double sign 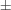 in front of the third term on the right-hand side, which gives indefiniteness by π to the phase of the cosine function. Which of the two signs ‘
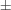
’ should be taken was determined by comparing the intensity calculated by equation (39) with that by the original equation (20). From the result of thus checking the phases of many moiré fringes, it was found that the correct sign to be adopted switches alternately with a period of some spatial interval. Another, more important, problem in equation (39) is a discontinuous change by ±π in the phase term 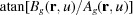. This discontinuous change occurs in two ways: one is when 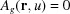 and 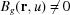, and another is when 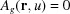 and 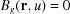. The first type of phase jump occurs owing to the limits by the defined domain 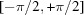. Such phase jumps are false jumps which do not occur in the original equation (20). In the calculations shown later, such false jumps were corrected manually one by one, and the correctness of the entire corrected phase was checked by comparing the intensities calculated by equations (39) and (20). This phase correction is analogous to the work of the atan2 function. However, we do not use atan2 here, since the corrected phase values are not necessarily held within the defined domain 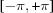. The second type of phase jump when 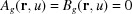 is a real phase jump, which also occurs in the calculation by equation (20). It is not difficult to confirm that 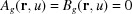 actually occurs when 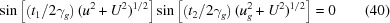
holds in equations (23*a*)[Disp-formula fd23a], (23*b*)[Disp-formula fd23b] for 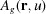 and 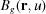, in the case of zero absorption (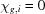). Then an abrupt change by 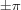 of the phase 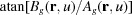 occurs for 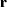 of 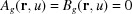. The reason for this can be understood from Fig. 5 ▸. When 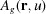 and 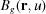 change sign at the same time as they pass through the origin, the value of 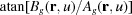 discontinuously changes by 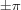 with the value of 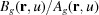 remaining unchanged. Though it is only in the extreme case of 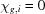 that 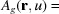 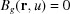 holds exactly, cases where 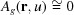 and 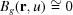 ordinarily occur. Then an analogous abrupt but continuous phase change occurs owing to the abrupt change of 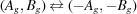. The position of the fringe changes abruptly as a result of this abrupt phase change. We call this abrupt change of half a period an abrupt fringe jump in this paper. This property of diffraction moiré fringes was not known at the start of this study, and was found in the search for the cause of the local bending of moiré fringes. The phase 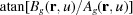 is given as 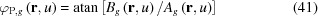
[the sign is reversed compared to the definition in Yoshimura (1996*a* ▸)]. From equations (39) and (37*c*), the equation for the fringe line (*i.e*., the equi-phase line) when 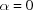 is given by 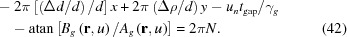
In what follows we present examples of moiré images showing abrupt fringe jumps, computed under several different sets of conditions. All the images were computed using the original equation (20), not by equation (39). Visual Basic .NET, version 2003, was used to write the computation. In all the computations the Si 220 reflection with Mo *K*α radiation (0.070926 nm) was assumed; θ_B_ = 10.64°; the symmetric Laue geometry (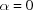, 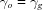) was assumed. To avoid complications when interpreting the computed images, the fringe pattern was assumed to be of rotation moiré with (Δ*d*/*d*) = 0, except for the case in Fig. 13. The relative rotation of the diffracting plane for the rotation moiré was assumed to be Δρ = *d* /0.045 (rad), to make the fringe spacing Λ = 0.45 mm (*d* = 0.19202 nm). The front crystal *A* of the bicrystal was assumed to be strain-free except for the case in Fig. 12(*a*). The rear crystal *B* was assumed to be lightly curved around the *y* axis concavely in the outward direction, with a curvature of *s* = 0.045′′ per mm. This causes an inclination of the diffracting lattice plane that is given by 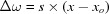 (*x*_o_ = 9.0 mm). The thickness of the interspacing air gap was assumed to be *t*_gap_ = 0.024 mm. Deviation parameters in equation (11) and equations (16*a*), (16*b*) are calculated as 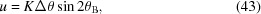


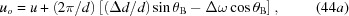


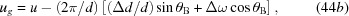
under the above assumption of symmetric Laue geometry.

**Figure 6:**
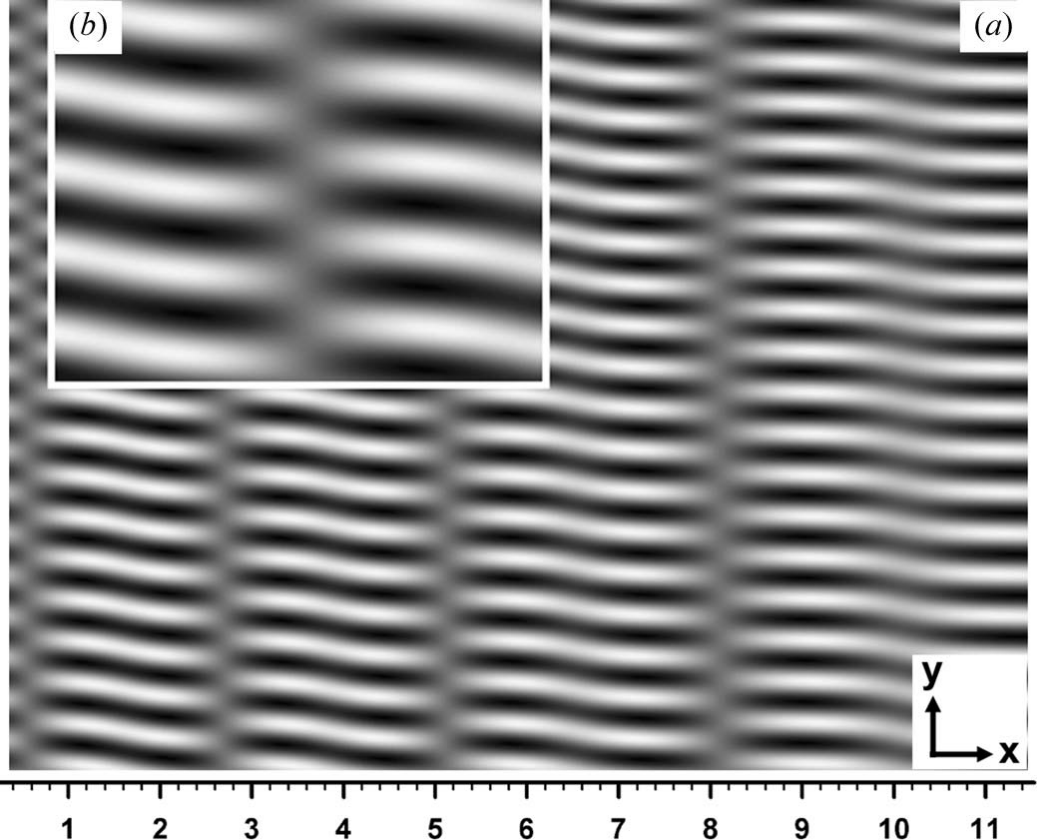
(*a*) Computer simulation of X-ray diffraction moiré image (rotation moiré of the fringe spacing 0.45 mm) with a silicon bicrystal assumed as the specimen. The 220 reflection with Mo *K*α radiation (0.070926 nm) was assumed; plane-wave *G* image with the deviation angle 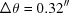; zero absorption (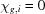) was assumed. Thicknesses of the component crystals of the bicrystal were 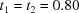
 mm, and that of the interspacing gap was 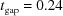
 mm. The rear component crystal *B* was assumed to be lightly curved with a curvature of 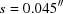 per mm. The scale in the *y* direction is the same as that in the *x* direction. See text for more details. (*b*) Doubly magnified image of the image in (*a*), to show fringe jumps and discontinuities in detail.

**Figure 7:**
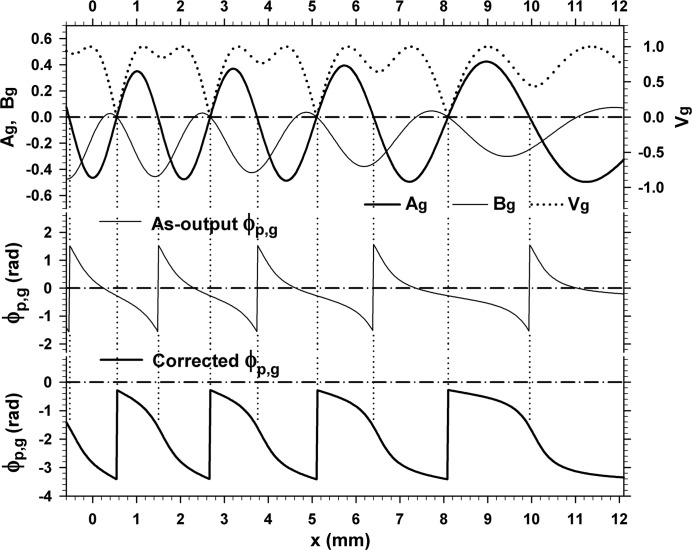
Top: curves of 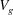 [fringe contrast, calculated by equation (20)], and of 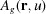 and 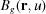 calculated by equations (23*a*)[Disp-formula fd23a], (23*b*)[Disp-formula fd23b]. Middle: curves of the PL phase as calculated by equation (41). Bottom: curves of the corrected PL phase. All the graphs are for the moiré image in Fig. 6 ▸. See text for details.

**Figure 8:**
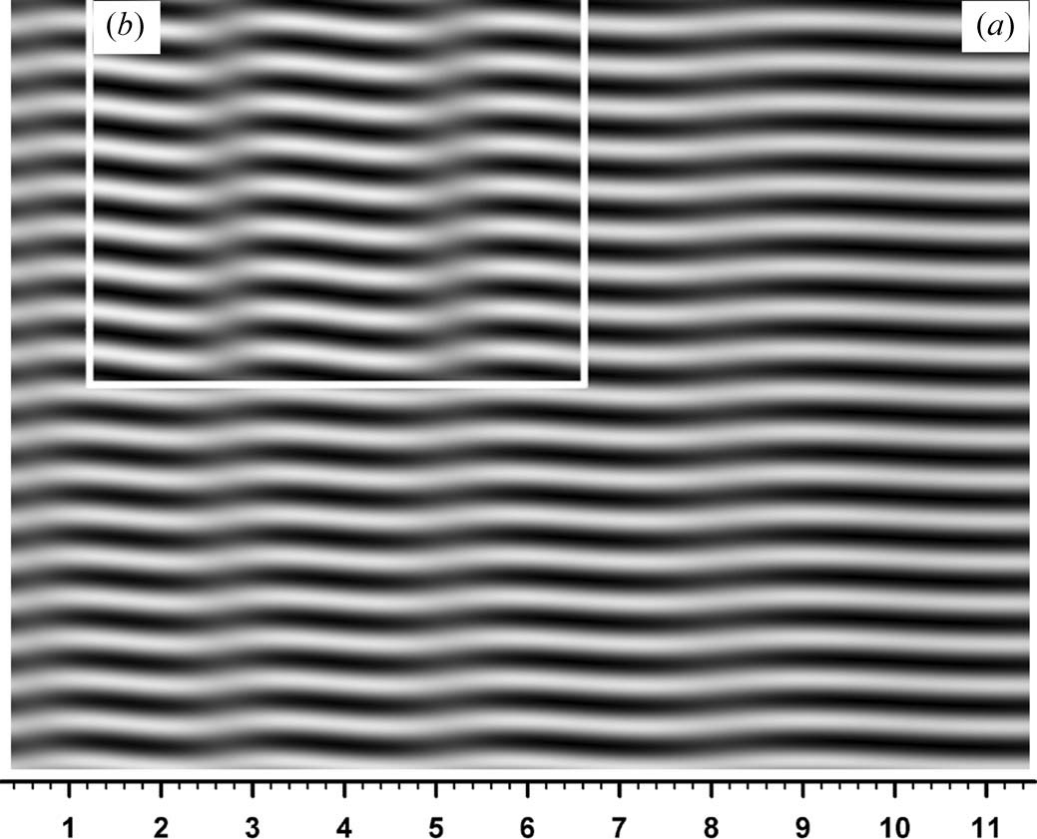
Moiré images computed under the same conditions as for Fig. 6 ▸, except for the value of crystal absorption. (*a*) Image when the real value of absorption was used; (*b*) image when half the real value was assumed.

**Figure 9:**
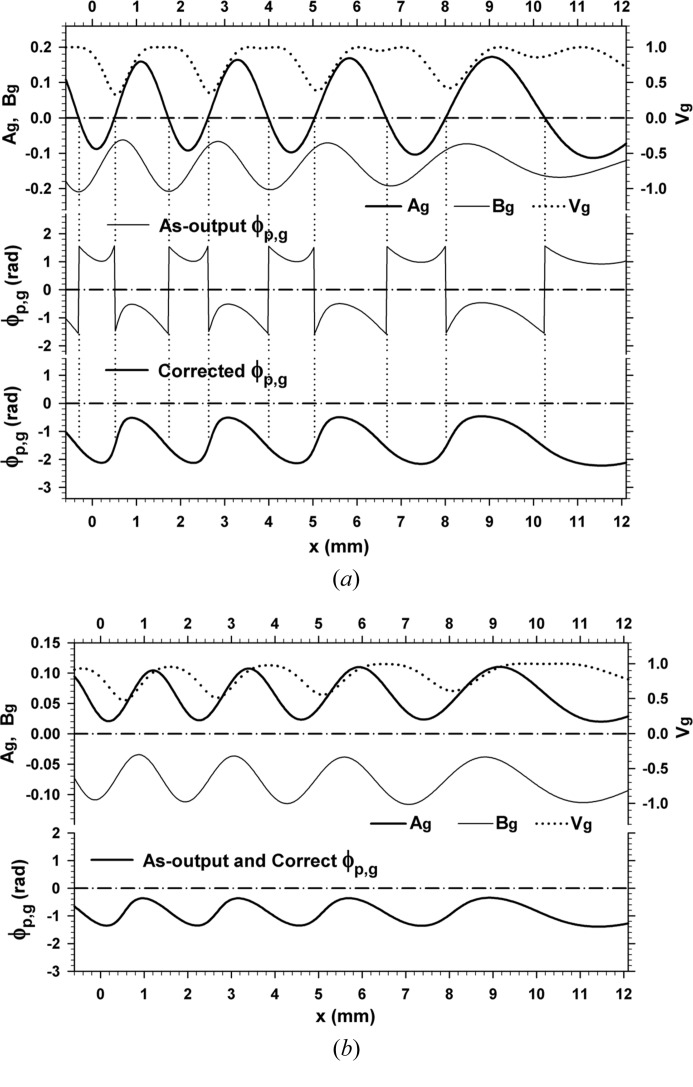
Calculated curves of 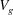, 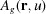 and 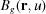, and of the as-calculated and the corrected PL phases, analogous to Fig. 7 ▸. The graphs in (*a*) are for the moiré image in Fig. 8 ▸(*b*) (inset), and those in (*b*) are for the moiré image in Fig. 8 ▸(*a*) (main figure).

**Figure 10:**
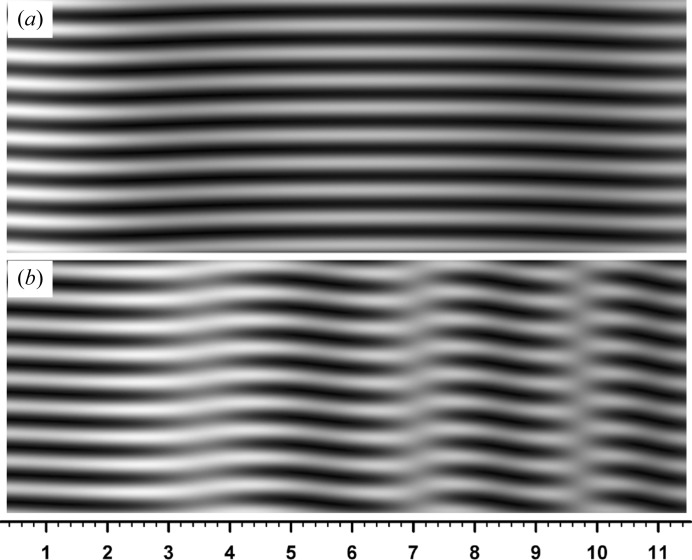
Computed moiré images under the same conditions as for Fig. 8 ▸(*a*), but with (*a*) 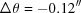 and (*b*) 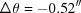.

First, Fig. 6 ▸ shows a computed moiré image when crystal absorption was imaginarily assumed zero (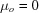, 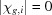). The crystal thicknesses and the deviation angle were assumed to be 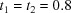
 mm and 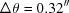. Although opposite to the main convention, the images in this paper are presented so that white contrast indicates higher intensity. The aforementioned abrupt fringe jumps can be observed at 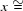 0.6, 2.7, 5.1 and 8.1 mm. The fringe jumps in this case are exactly the half-a-period positional change, and fringe lines are discontinuous between facing image regions. The magnified image in the inset shows details of the fringe jumps and discontinuity. Fig. 7 ▸ shows the curves of 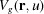 (fringe contrast), 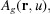 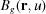 and 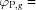 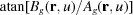 calculated by equations (20), (23*a*)[Disp-formula fd23a], (23*b*)[Disp-formula fd23b] and (41), for the moiré image in Fig. 6 ▸. The curve of 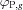 in the middle figure by equation (41) is corrected to the curve as in the bottom figure, in the way described earlier. Fringes in Fig. 6 ▸ change their position in accordance with this corrected phase curve, on the basis of the fringe-line equation (42). Discontinuous 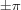 phase jumps are clearly recognized in this phase curve. Fig. 8 ▸ shows a moiré image computed with the real value of absorption, with other conditions being the same as those for Fig. 6 ▸. However, since the image is much changed from that in Fig. 6 ▸, an image imaginarily computed with half the real value of absorption is appended in the inset in Fig. 8 ▸(*b*). (The change in the 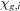 value for absorption by changing the wavelength also causes an unwanted change in 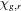, making an easy comparison difficult.) Fig. 9 ▸(*a*) shows the calculated curves of 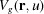, 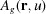, 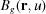 and 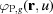 for the inset image, Fig. 8 ▸(*b*). Although the condition 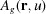 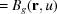 = 0 no longer holds, abrupt changes of the phase 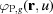 approximately occur where 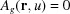 and 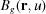 comes close to zero. Fig. 9 ▸(*b*) shows calculated curves of 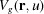, 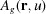, 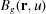 and 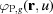 for the image in the main figure, Fig. 8 ▸(*a*). For this image, the condition 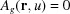 nowhere holds, and phase jumps do not occur. Nevertheless, oscillations of 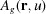, 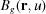 and 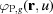 occur though not an abrupt change, and the fringes undulate correspondingly. Figs. 10 ▸(*a*), 10 ▸(*b*) show moiré images at 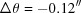 and 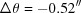 for comparison with the image in Fig. 8 ▸(*a*) at 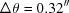; they were computed with all conditions other than 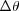 taken to be the same as for Fig. 8 ▸(*a*). As can be seen in the three images, when the angular position (*i.e*., deviation angle) 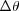 varies from the positive to negative side, the fringes become nearly flat in the vicinity of 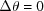; as 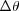 further goes on in the negative region, the fringes begin to undulate again with a short interval.

**Figure 11:**
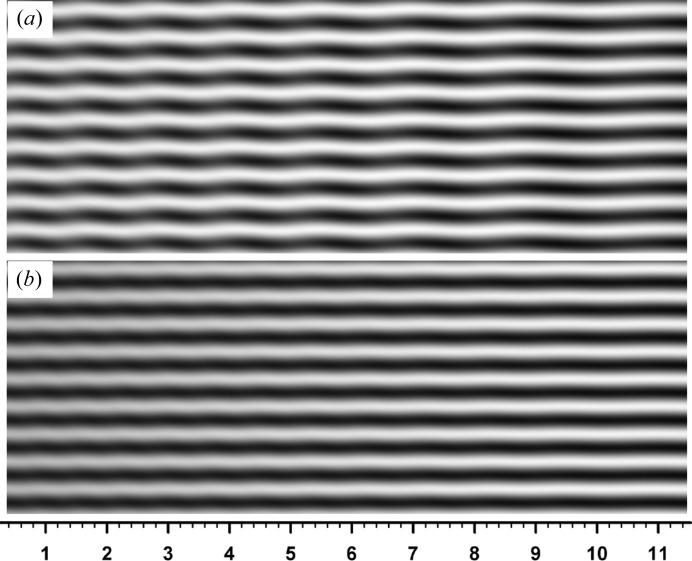
Computed moiré images under the same conditions as for Fig. 8 ▸(*a*), but with the crystal thicknesses of (*a*) 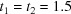
 mm and (*b*) 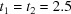
 mm.

Figs. 11 ▸(*a*), 11 ▸(*b*) show moiré images computed for crystal thicknesses 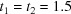 and 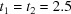 (mm), respectively, with the deviation angle being the same as for Fig. 8 ▸(*a*), *i.e.*, 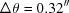; the real value of absorption was used. As seen in these images, the amplitude of the fringe undulation gradually decays while the oscillation interval becomes shorter, with the increasing crystal thicknesses. The undulation is still seen at 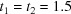
 mm, but almost disappears at 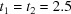
 mm. Such decay of the fringe undulation is analogous to the decay of subsidiary maxima and minima with increasing absorption, in a rocking curve showing an equi-inclination oscillation. (The 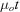 values are 2.2 for 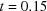
 mm, and 3.7 for 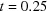
 mm.)

**Figure 12:**
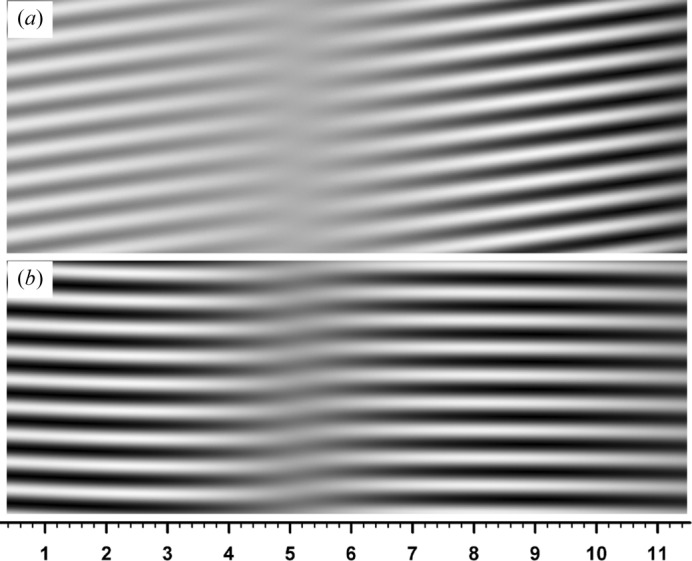
Computed moiré images under the same conditions as for Fig. 8 ▸(*a*), but with the crystal thicknesses of (*a*) 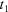 = 0.2, 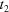 = 2.0 (mm) and (*b*) 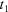 = 2.0, 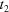 = 0.2 (mm). [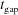 = 0.24 mm and 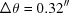 in both (*a*) and (*b*).] For the image (*a*), the front crystal *A* is assumed to be strained, unlike the assumption for other computed images. See text for more details.

Figs. 12 ▸(*a*), 12 ▸(*b*) show examples of when either of crystals *A* and *B* is thin. The image in Fig. 12 ▸(*a*) was computed assuming that the thin front crystal *A* (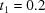
 mm) is curved concavely in the outward direction, and the thick rear crystal *B* (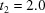
 mm) is strain-free. In this case only, where crystal *A* is strained, the following equations were employed for the calculation of *u*, 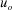 and 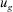: 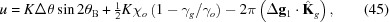


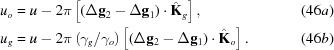
Here, 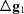 is a local change in the reciprocal-lattice vector from 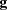 for the perfect region in crystal *A*; 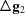 is a change in the reciprocal-lattice vector in crystal *B*, relative to the same reciprocal-lattice vector 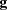 in crystal *A*; 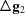 is the same as the previously used 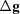. The inclination of the diffracting plane in crystal *A* was given as 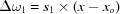 with 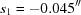 per mm (*x*_*o*_ = 9.0 mm), while that in crystal *B* was 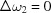; 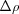 and (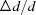) were the same as in Fig. 8 ▸(*a*); 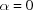. Then, *u*, 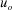 and 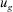 in the above equations become 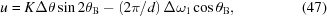


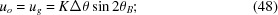
the deviation angle was 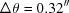. In the computed image an abrupt fringe jump (or a local bending of fringes) as observed in Fig. 8 ▸ *etc*. can be seen around 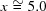. The fringes are inclined as if the intrinsic moiré pattern has a parallel component (Δ*d*/*d*), but that is in reality due to the contribution from the gap phase 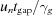, which varies with *x*, being caused by the variation of *u* in equation (47). The abrupt fringe jump is caused by the curvature in crystal *A*. Since 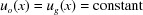 in this case, crystal *B* takes no part in the fringe jump. The assumption that crystal *A* is thin is no essential condition. Also for thicker crystal *A*, abrupt fringe jumps would occur analogously to Fig. 8 ▸ *etc*., though fringes are inclined owing to the phase 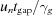. A description that appears to refer to a similar effect of the gap phase can be found in Tanemura & Lang (1973 ▸). The image in Fig. 12 ▸(*b*), for the case that the rear crystal *B* is thin (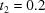
 mm), was computed under the same conditions as for Fig. 8 ▸(*a*) except for the crystal thicknesses, and with equations (43) and (44*a*)[Disp-formula fd44a], (44*b*)[Disp-formula fd44b] used again for the calculation of *u*, 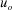 and 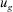; crystal *A* was assumed to be strain-free, while crystal *B* was assumed to have curvature *s* = 0.045′′ per mm; 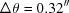. When the crystal becomes thin, the crystal absorption is smaller and the fringe jumps should be more clearly abrupt. However, on the other hand, related variables such as 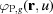 vary more slowly, so that fringe jumps become more gently sloped and widely spaced.

In the above, we have surveyed how abrupt fringe jumps appear depending on the values of 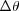, 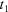, 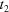 and the magnitude of absorption, with 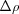 and *s* being fixed and 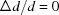. As the curvature |*s*| becomes larger, the number of abrupt fringe jumps increases, but the height of the jumps does not significantly change since it is determined by the magnitude of absorption. When the sense of curvature becomes opposite (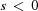), the bending of fringes becomes of a shape symmetric to that in Fig. 8 ▸ *etc*., as a broad outline. Namely, when 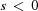, the fringe position (*y*) slowly rises from left to right and is abruptly lowered. (Such fringe bending in plane-wave images differs from that in integrated intensity images.) When the diffracting lattice plane is uniformly inclined (about the *y* axis) without curvature (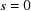), namely, 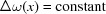, the uniform inclination 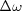 does not affect the fringe pattern since 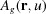 and 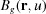 do not vary with the position in the crystal. Furthermore, so far as the diffracting plane is exactly parallel to the surface normal 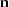 (*i.e.* 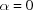), the inclination 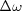 does not affect the intrinsic moiré pattern, as can be seen from equation (37*b*). Because of a uniform change in 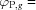 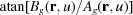 by the uniform inclination 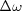, the entire fringe pattern is uniformly displaced by a corresponding distance in the *x* and/or *y* directions, in accordance with equation (42).

**Figure 13:**
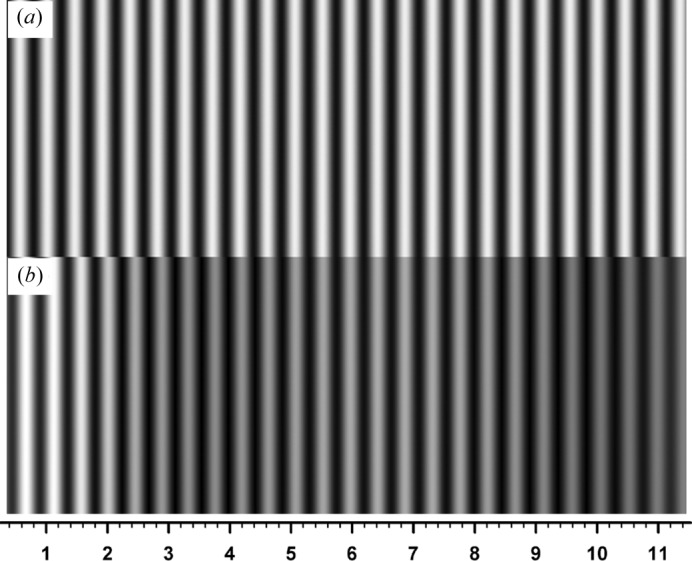
Computed moiré images for a parallel moiré with 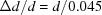 and 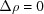. The crystal and gap thicknesses were the same as for Fig. 8 ▸(*a*). 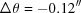. (*a*) Moiré image when both crystals *A* and *B* have no local strain. (*b*) Moiré image when crystal *B* has a curvature of 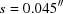 per mm around the *y* axis, similar to the case of Fig. 8 ▸(*a*) *etc*. See text for more details.

**Figure 14:**
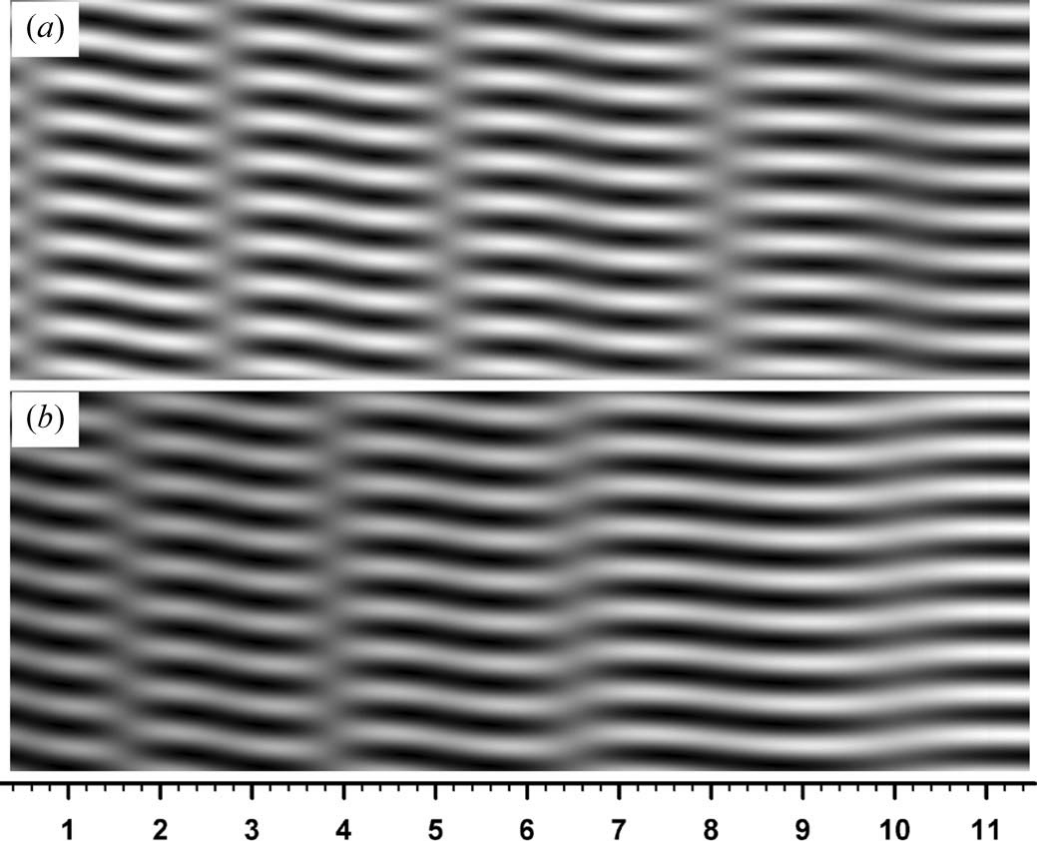
Moiré images of transmitted wave (*O* wave), computed under the same conditions as for Figs. 6 ▸ and 8(*a*) ▸ regarding the values of 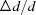, 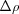, the crystal and gap thicknesses and the crystal curvature; 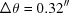. (*a*) Moiré image when zero absorption was assumed. (*b*) Moiré image when the real value of absorption was used.

**Figure 15:**
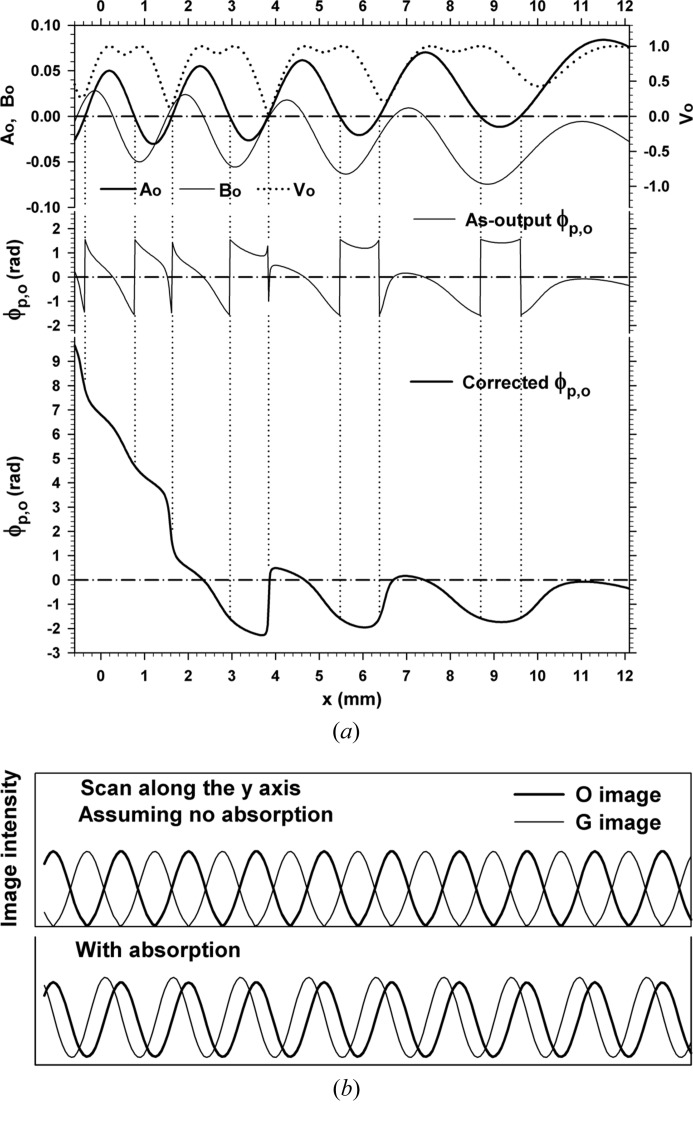
(*a*) Calculated curves of 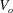 (fringe contrast), 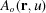, 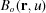 and of the as-calculated and the corrected PL phases, associated with the moiré image in Fig. 14 ▸(*b*). (*b*) Intensity profiles by the scan along the *y* axis in the computed moiré images, for the comparison between the *O* and *G* images. The top figure compares the profiles in Fig. 6 ▸ and Fig. 14 ▸(*a*) for the case that zero absorption was assumed; the bottom figure compares profiles in Fig. 8 ▸(*a*) and Fig. 14 ▸(*b*) when the real value of absorption was used.

Figs. 13 ▸(*a*), 13 ▸(*b*) show moiré images computed for a parallel moiré of 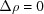 and 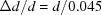, with the assumption of no curvature (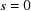) and the curvature of *s* = 0.045′′ per mm in crystal *B*, respectively. The deviation parameters *u*, 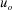 and 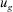 were calculated by equations (43) and (44*a*)[Disp-formula fd44a], (44*b*)[Disp-formula fd44b] in the same way as for Fig. 8 ▸(*a*). Though not so large as to be easily noticed without close comparison, the fringe spacing in Fig. 13 ▸(*b*) is modified relative to that in Fig. 13 ▸(*a*). The image intensity in Fig. 13 ▸(*b*) is also considerably modified compared with that in Fig. 13 ▸(*a*). (The intensity modulation was large and rapid at 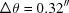, and therefore the images at 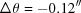 of a weaker modulation are presented.) Thus, the combined effect of the crystal curvature and the PL phase can also be seen in such differences between the two images of parallel moiré. Finally, an example of moiré images of the *O* wave is shown in Figs. 14 ▸(*a*), 14 ▸(*b*), although the associated intensity equation was omitted. The computation was conducted using the same values of 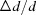, 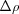, *s*, 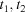, 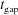 and 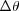, as for Fig. 8 ▸(*a*). Fig. 14 ▸(*a*) shows the image when zero absorption was assumed and is to be compared with Fig. 6 ▸. Fig. 14 ▸(*b*) was computed with the real value of absorption, and is compared with Fig. 8 ▸(*a*). Calculated curves of fringe contrast 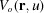 and of phase-related variables 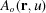, 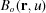 and 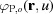 associated with the image in Fig. 14 ▸(*b*) are shown in Fig. 15 ▸(*a*). [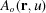, 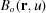 and 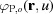 correspond to 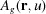, 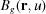 and 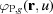 for the *G* image, respectively.] The fringe pattern in Fig. 14 ▸(*a*) is almost the same as that in Fig. 6 ▸, but the fringe position is displaced by half a period, as shown in the top figure in Fig. 15 ▸(*b*). As the intensity profiles of these two fringe patterns show, the complementarity of diffracted intensities between the *O* and *G* images holds in this case. When absorption has the real value, vertical bands of abrupt fringe jumps in the *O* and *G* images are displaced from each other by nearly half the interval, as can be seen in Fig. 8 ▸(*a*) and Fig. 14 ▸(*b*). On the other hand, their fringe positions come nearer to each other, as shown in the bottom figure in Fig. 15 ▸(*b*). A comparison of experimental *O* and *G* images of moiré fringes in such a relation has been presented in Yoshimura (1997*a* ▸).

## 5. Summary   

The theory of X-ray diffraction moiré fringes with a bicrystal specimen has been described by plane-wave dynamical diffraction theory. In the development of the theory, attention was paid to describing the double diffraction of moiré interference exactly and in detail. On the basis of the developed theory, the effect of crystal strain and *Pendellösung* intensity oscillation on the interference pattern of moiré fringes was studied in detail with the theoretical calculations of the moiré image and of the phase-related variables. Through this work, it was revealed that crystal diffraction moiré fringes have the basic property of an abrupt fringe jump of half a period. It was found that, when the front crystal of a bicrystal is strained, significant modification to the moiré fringe pattern can occur owing to the local variation of the gap phase caused by the strain. Furthermore, a pending question for a long time regarding the dimensionality of crystal diffraction moiré fringes has been settled. A theoretical study of the integrated intensity image of moiré fringes will be given elsewhere, following this theory of the plane-wave image.
